# Age-specific haemosporidian infection dynamics and survival in Seychelles warblers

**DOI:** 10.1038/srep29720

**Published:** 2016-07-19

**Authors:** Martijn Hammers, Jan Komdeur, Sjouke A. Kingma, Kimberly Hutchings, Eleanor A. Fairfield, Danielle L. Gilroy, David S. Richardson

**Affiliations:** 1 Behavioural and Physiological Ecology, GELIFES, University of Groningen, P.O. Box 11103, 9700 CC, Groningen, The Netherlands; 2 Theoretical Research in Evolutionary Life Sciences, GELIFES, University of Groningen, P.O. Box 11103, 9700 CC, Groningen, The Netherlands; 3 School of Biological Sciences, University of East Anglia, Norwich Research Park, Norfolk, NR4 7TJ, UK; 4 Nature Seychelles, Roche Caiman, Mahé, Republic of Seychelles

## Abstract

Parasites may severely impact the fitness and life-history of their hosts. After infection, surviving individuals may suppress the growth of the parasite, or completely clear the infection and develop immunity. Consequently, parasite prevalence is predicted to decline with age. Among elderly individuals, immunosenescence may lead to a late-life increase in infection prevalence. We used a 21-year longitudinal dataset from one population of individually-marked Seychelles warblers (*Acrocephalus sechellensis*) to investigate age-dependent prevalence of the GRW1 strain of the intracellular protozoan blood parasite *Haemoproteus nucleocondensus* and whether infections with this parasite affect age-dependent survival. We analyzed 2454 samples from 1431 individuals and found that *H. nucleocondensus* infections could rarely be detected in nestlings. Prevalence increased strongly among fledglings and peaked among older first year birds. Prevalence was high among younger adults and declined steeply until *ca* 4 years of age, after which it was stable. Contrary to expectations, *H. nucleocondensus* prevalence did not increase among elderly individuals and we found no evidence that annual survival was lower in individuals suffering from an infection. Our results suggest that individuals clear or suppress infections and acquire immunity against future infections, and provide no evidence for immunosenescence nor an impact of chronic infections on survival.

Parasites can have severe impacts on the fitness of their hosts, and can influence reproduction^1^, survival^2^ and sexual selection^3^. Therefore, understanding if and how hosts mitigate the negative effects of infections is of major importance to understanding host-parasite co-evolution and host life-history evolution^4^.

Among the several groups of parasites that infect birds, haemosporidian blood parasites of the genus *Haemoproteus* are common^5^. Infections with *Haemoproteus sp.* are characterized by several stages^5^. After transmission from the insect vector to the vertebrate host, sporozoites develop within host cells, where they reproduce asexually and form pre-erythrocytic meronts. The merozoites released by these meronts enter circulating red blood cells, where they develop into mature gametocytes. Subsequently, the intensity of the infection increases until the parasites occur in a substantial proportion of the blood cells (the acute stage of the infection). Individuals that survive this acute stage may develop immune responses, suppress the infection and acquire immunity against the parasite, leading to rapid declines in infection intensity and recovery of the host. However, under natural conditions, low levels of *Haemoproteus* parasites (and other types of haemosporidian parasites) may survive for a long time in host tissues after an infection, and relapses of infections may occur^5^. Also, re-infections with the same or other *Haemoproteus* strains may occur.

One factor explaining variation in *Haemoproteus* prevalence among individuals is age. In birds, *Haemoproteus* infections are rarely detected in red blood cells in nestlings and young fledglings^6^,^7^, probably because the time between an infection being transmitted to a nestling and *Haemoproteus* parasites entering the blood stream is often longer than the nestling stage^8^. After contracting an infection, individuals may become ill and die during the acute phase, or develop an effective immune response and suppress or clear the infection. Consequently, the probability that haemosporidian parasites can be detected in red blood cells may decline with age as older individuals will be more likely to have been exposed to the parasite, survived, and developed an immune response. Conversely however, an age-dependent deterioration of the immune system (immunosenescence^9^,^10^) might lead to a late-life increase in (re-) infection probability in elderly, uninfected individuals, the inability to suppress already existing latent infections, or greater costs of infection in elderly compared to mid-aged individuals (see ref. 11). Higher parasite prevalence and higher intensity of infections among elderly individuals may lead to decreased late-life reproduction and increased late-life mortality, potentially serving as a mechanistic explanation contributing to senescence patterns.

Several previous studies in natural populations have shown age-dependence of *Haemoproteus* infection among adults, including age-dependent increases^12^,^13^ or decreases^14^. However, such studies often include few elderly individuals, or do not sample individuals repeatedly throughout their lives (e.g. ref. 15). Consequently, it remains unclear whether *Haemoproteus* prevalence increases in elderly individuals as a result of immunosenescence, or whether changes in prevalence in relation to age are the result of biased mortality in cross-sectional studies. Furthermore, little is known about whether *Haemoproteus* infections affect age-dependent patterns of survival.

Here, we investigate age-dependent *Haemoproteus* prevalence and the relationship between *Haemoproteus* infection and age-dependent survival in the Seychelles warbler (*Acrocephalus sechellensis*) population on Cousin Island. This population is ideally suited for this purpose as most individuals (>95%) are individually marked, measures of survival are not confounded by dispersal off the island^16^, and individuals live long enough to show reproductive and survival senescence, at least partly due to the lack of adult predation (reviewed in^17^). Since 1994, many individuals have been repeatedly blood sampled (and screened for haemosporidian parasites) over their lifespan. In many species, individuals may be infected with multiple lineages or species of haemosporidian blood parasites^18^,^19^,^20^,^21^, which may complicate the study of age-dependent prevalence and the fitness consequences of infections^22^. In the Seychelles warbler, however, only one blood parasite has been detected despite extensive screening over many years; the GRW1 lineage of the haemosporidian parasite *Haemoproteus nucleocondensus*^23^ (formerly known as *Haemoproteus payevskyi*^24^). The occurrence of a single parasite strain makes the Seychelles warbler a tractable system in which to study the age-dependent prevalence and fitness consequences of (at least this strain of) *Haemoproteus* blood parasites.

A previous microscopy study on *H. nucleocondensus* in the Seychelles warbler found that prevalence in adults declined with age, that males were more likely to be infected than females, and that infected juveniles showed lower survival compared to uninfected juveniles^14^. However, this previous study used blood smears, which may be less sensitive at detecting *Haemoproteus* infections than the nested PCR (polymerase chain reaction) method used in the present study. Moreover, that study only investigated a small number of individuals (*n *= 50) with only 20 individuals measured on multiple (two) occasions, making it difficult to draw conclusions about within-individual changes in age-dependent infection prevalence. Finally, as few individuals were older than the average lifespan of Seychelles warblers (mean lifespan 5.5 years^25^), the authors were not able to study late-life patterns of infection prevalence and survival.

In this study, we measured the prevalence of *H. nucleocondensus* over the lifespan of Seychelles warblers using a nested PCR method^26^. We screened a total of 2454 blood samples collected between 1994 and 2014 from 1431 individuals and used the results in conjunction with accurate and extensive survival data available for this population to investigate whether infection by *H. nucleocondensus* in the Seychelles warbler 1) shows age-dependent prevalence, and 2) affects survival.

## Results

### Age-dependent *Haemoproteus* prevalence

**Figure 1:**
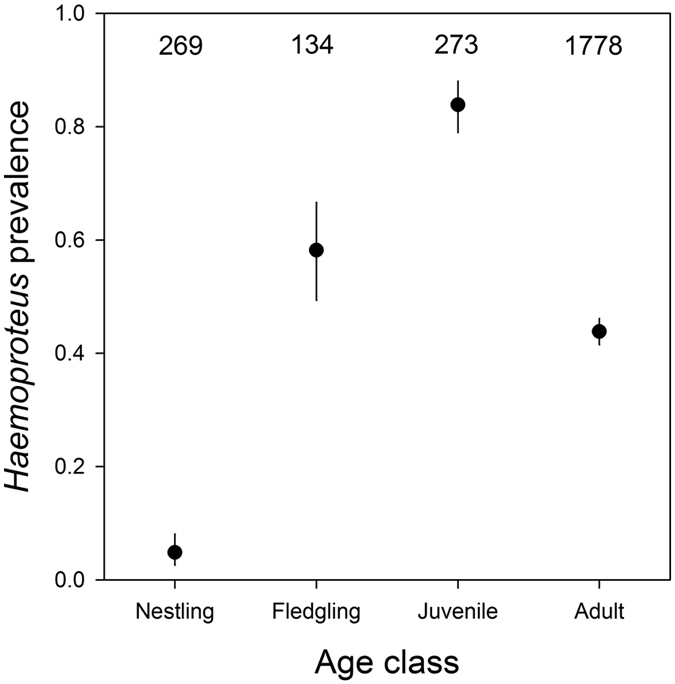
*Haemoproteus* prevalence in different age-classes. Prevalence of infections with the GRW1 strain of *Haemoproteus nucleocondensus* in Seychelles warblers of different age classes. Data are means and 95% confidence intervals per age class. Numbers represent sample sizes.

The prevalence *H. nucleocondensus* differed strongly between the four age classes (Tukey contrasts: all *P* < 0.001; Fig. 1). As expected, infections were rarely detected in nestlings (5%, 13 out of 269 samples), but infection prevalence was much higher in fledglings (58%, 78 out of 134 samples) and highest among juveniles (84%, 229 out of 273 samples). Prevalence in juveniles differed between cohorts (*Χ*^2^* *=* *61.03, *df *=* *20, *P* < 0.001) and ranged from 29 to 100%. In adults, we detected infections in 44% (779 out of 1778) of samples.

**Figure 2:**
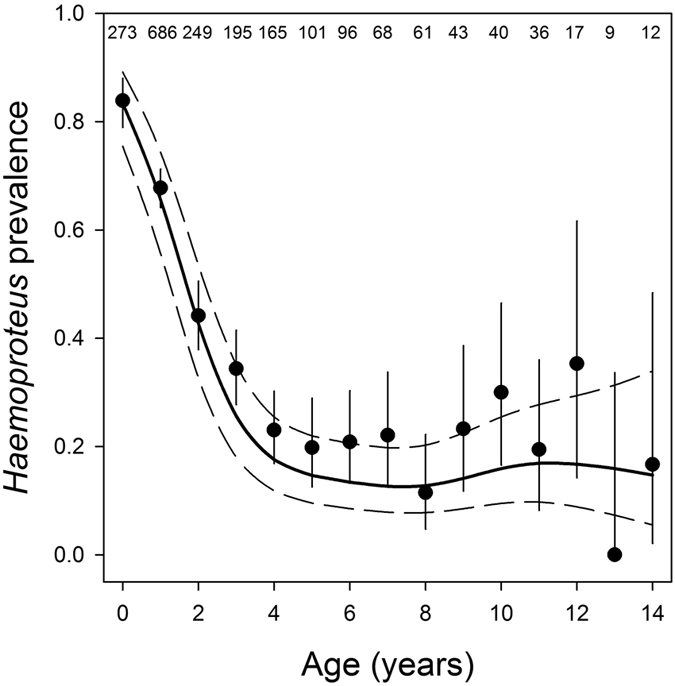
Age-dependent *Haemoproteus* prevalence. Prevalence of infections with the GRW1 strain of *Haemoproteus nucleocondensus* in Seychelles warblers in relation to age. The solid line shows the model predicted regression slope from a GAMM with a non-parametric smoothing function for age and the dashed lines are 95% confidence intervals. Data are means and 95% confidence intervals for each age. Nestlings and fledglings are not included in the 0 year age class and are presented in Fig. 1. Ages 14–17 are grouped for graphical purposes (denoted as 14 here). Numbers represent sample sizes.

The GAMM analysis, which included a significant non-parametric smoothing function for age (*P* < 0.001, *df *=* *4.76), showed that infection prevalence declined strongly with age until *ca* 4 years, stabilising at a consistent low level from *ca* 5 years onwards (Fig. 2). We found no evidence that shorter-lived individuals had higher infection prevalence than longer lived individuals (i.e. no selective disappearance; β lifespan ± SE* *=* *−0.03 ± 0.03, z* *=* *−1.15, *P *=* *0.25). Males tended to have higher infection prevalence than females, but this difference was not significant. (β ± SE* *=* *0.20 ± 0.12, z* *=* *1.69, *P *=* *0.09).

Consistent with the GAMM analysis outlined above, the analysis separating within- and between individual age effects showed that infection prevalence declined strongly with age in individuals ≤4 years old, both at the population level and within individuals (Table 1). The slopes of the within- (longitudinal) and between-individual (cross sectional) declines in infection prevalence were similar (β mean age - β delta age ± SE* *=* *0.16 ± 0.16, z* *=* *0.98, *P *=* *0.33). Both sexes showed similar age-dependent declines in infection prevalence (the interaction between delta age and sex was not significant, Table 1).

In individuals ≥7 years of age, infection prevalence did not change with age; the slopes of the cross-sectional and within-individual declines in infection prevalence with age were not significantly different from zero (Table 1) and did not differ from each other (β mean age - β delta age ± SE* *=* *0.19 ± 0.19, z* *=* *0.98, *P *=* *0.33).

**Figure 3:**
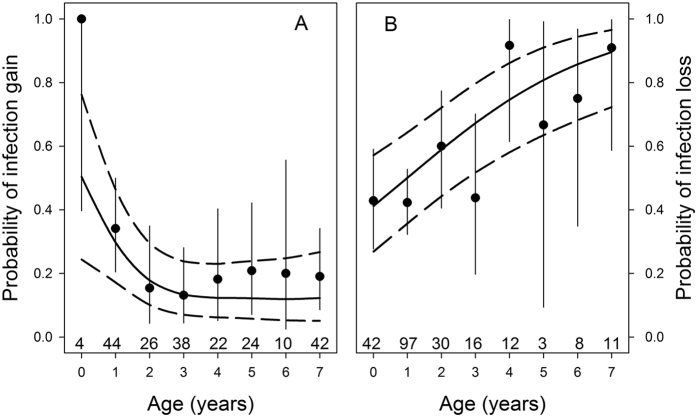
Age-dependent gain and loss of *Haemoproteus* infections. The probability that (**A**) individuals that tested negative for infection with the GRW1 strain of *Haemoproteus nucleocondensus* in a given year tested positive in the next year, and conversely (**B**) individuals that tested positive in a given year tested negative the next year, in relation to age. The solid line shows the model predicted regression slope from a GAMM with a non-parametric smoothing function for age. The dashed lines are 95% confidence intervals. Data are means and 95% confidence intervals. Ages 7–17 are grouped for graphical purposes (denoted as 7 here). Numbers represent sample sizes.

In 63% (269 out of 429) of individuals that were sampled in two consecutive years, infection status did not change. Individuals appeared more likely to clear or suppress an infection to below detectable levels than to gain it or suffer from a relapse from a previous infection: 22% (47 out of 210) of individuals that tested negative in the first year tested positive in the next year and 52% (113 out of 219) of individuals that tested positive in the first year tested negative in the subsequent year. The probability that individuals that tested negative in the first year were diagnosed with an infection in the next year was highest among individuals younger than a year (4 out of 4 individuals) and was relatively constant in older individuals (Fig. 3, GAMM: *P* < 0.05, *df *=* *3.41). The probability that individuals that tested positive in the first year tested negative in the second year increased with age (Fig. 3, GAMM: *P* < 0.001, *df *=* *1). There was no difference between the sexes in both analyses (*P* > 0.33).

### *Haemoproteus* infection and age-dependent survival

Considering individuals of all ages, survival to the next year was not lower in individuals that tested positive for *H. nucleocondensus* (β ± SE* *=* *−0.03 ± 0.14, z* *=* *−0.24, *P *=* *0.81) and survival was not predicted by sex (β ± SE* *=* *−0.11 ± 0.12, z* *=* *−0.90, *P *=* *0.37) or age (GAMM: *P *=* *0.55, *df *=* *1).

In individuals ≤4 years old, age-dependent survival probability did not differ significantly between individuals that were observed to carry an infection or not (interaction between age and infection status: β ± SE* *=* *0.23 ± 0.13, z* *=* *1.81, *P *=* *0.07). This non-significant interaction term might show that survival of individuals with detectable levels of the parasite increases with age, whereas survival remains relatively constant for uninfected individuals or individuals with very low intensity or suppressed infections. While survival probability tended to increase with age (β ± SE* *=* *0.12 ± 0.06, z* *=* *1.84, *P *=* *0.07), infection status (β ± SE* *=* *0.07 ± 0.16, z* *=* *0.46, *P *=* *0.65) and sex (β ± SE* *=* *−0.11 ± 0.14, z* *=* *−0.77, *P *=* *0.44) did not explain survival. In individuals ≥7 years old none of these variables were significant (all *P* > 0.27).

When we investigated annual survival separately in the age groups that are most likely to suffer from infections (juveniles and one year old individuals), we also found no difference in survival probability in infected and uninfected individuals (juveniles: β ± SE* *=* *−0.42 ± 0.50, z* *=* *−0.84, *P *=* *0.40; one year olds: β ± SE* *=* *0.08 ± 0.23, z* *=* *0.34, *P *=* *0.73).

## Discussion

Our results show that prevalence of the GRW1 strain of the haemosporidian parasite *Haemoproteus nucleocondensus* in Seychelles warblers decreases until *ca* 4 years of age and then remains stable. We found no evidence of increased infection prevalence during late life and we detected no clear effect of infection on survival. In the Seychelles warbler, most individuals probably become infected during the first year of their life. Most individuals are, at best, sampled once per year, therefore the 84% infection prevalence in juveniles is probably an underestimate of the total proportion of individuals that become infected during their first year, as some juveniles may be tested early in that period before being infected, while others are tested late in the period and have already suppressed or cleared the infection. The prevalence of infection among juveniles was much higher than the prevalence among adults of any age. The age-dependent decline in prevalence in adults most likely occurs because individuals suppress or clear infections, and was not caused by selective disappearance of infected individuals.

A previous study on the prevalence of *H. nucleocondensus* in the Cousin Island Seychelles warbler population also found a within-individual age-specific decline in prevalence and similar survival rates of adults that were scored as infected or uninfected^14^. The marginally significant (*P *=* *0.04) sex difference in infection prevalence (higher prevalence in males) shown by this previous study was not significant in this study (*P *=* *0.09), despite a more than 28-fold increase in sample size (50 vs 1431 individuals). The nested PCR technique we used to detect avian malaria distinguishes the presence or absence of the DNA of haemosporidian parasites circulating in the peripheral blood of the individual. Although this is a reliable method to detect the presence of haemosporidian parasites^27^, it provides no information about the intensity of the infection. The acute stage, where the intensity of the infection increases and peaks, is only brief, but sickness responses, such as reduced food intake and infection-related mortality, may occur mainly during these periods^28^,^29^. Individuals that suffer from an acute infection are likely to be underrepresented in our sampling as these individuals might be less likely to be caught using mist nets because, for example, these individuals are less active than chronically infected or uninfected individuals^5^,^8^. It seems likely that the infected individuals in this study, as in other studies using mist nets to catch individuals^20^, were those suffering from a chronic or latent infection, rather than the ones suffering from an acute infection. Thus, selective mortality may occur, but we may have failed to detect this because we only sampled individuals that survived the critical stages of infection. Similarly, failure to detect an infection may indicate that individuals are not infected, but an alternative possibility is that these individuals are infected but that the circulating levels of the parasite in the blood are too low to detect using the nested PCR method (although the nested PCR method is much more sensitive to low intensity infections than the microscopic examination of blood smears^27^,^30^). We have some indication that this may be the case here, as – in samples that tested positive for the parasite – the likelihood of a mismatch between two independent PCR screenings increased with age (binomial GLM: β ± SE* *=* *0.13 ± 0.03, *z *=* *4.26, *P* < 0.001). Low intensity infections may occur when individuals have acquired a certain amount of immunity against the parasite and can suppress the infection (e.g. ref. 31). During periods of stress, for example caused by low food availability or high reproductive investment (e.g. ref. 32), relapses of previously latent or chronic infections may occur and levels of the parasite in the blood might increase to levels that are detectable by the nested PCR method. To better understand these complications, future studies should not only investigate infection prevalence, but also the presence of antibodies (to check whether individuals have been infected previously) and infection intensity (e.g. using qPCR^13^,^21^,^27^).

Several studies have investigated changes in *Haemoproteus* prevalence with age, but the direction of this relationship is not consistent between studies^13^,^14^. As most Seychelles warblers probably become infected early in life (see Fig. 1 and above) and subsequently acquire immunity, suppress the infection, or die, the prevalence of *Haemoproteus* infections should decline with age, as was found in this study (until *ca* 4 years of age). Older individuals that tested positive for this parasite are thus most likely to be individuals that have not yet suppressed an infection to below detectable levels, are suffering from relapses of earlier infections, or have been re-infected. In the Seychelles warbler, only one strain of *Haemoproteus* has been detected, but in other species individuals are often infected with multiple haemosporidian parasites^18^,^19^,^20^,^21^. Prevalence of haemosporidian parasite infections may increase with host age in populations that harbour multiple parasite strains due to the increased likelihood of exposure of individuals to vectors (particularly as there may also be a greater range of potential vectors) or novel parasite lineages over time. For example, certain parasite strains may not occur in the breeding area, but individuals may become infected during migration or in their wintering area^33^, so that older individuals may accumulate more strains. In such cases, the age-dependent probability of becoming infected may be larger than the age-dependent loss of infections.

Although we found that individuals that tested negative for the presence of *H. nucleocondensus* in a given year could test positive in the subsequent year (either because they became re-infected or because the intensity of chronic infections increased to above the detection threshold), we did not find a late-life increase in *Haemoproteus* prevalence. Future studies may profitably investigate late-life *Haemoproteus* prevalence using a more detailed approach, such as determining the intensity of infection using qPCR, and investigate the role of environmental conditions and reproductive investment in determining infection intensity. In addition, future studies may focus on sampling individuals multiple times during the year in order to investigate changes in parasite prevalence over shorter time intervals and to increase the probability that an individual is sampled within a short period before its death, rather than just in the year before death.

We found no evidence that individuals that tested positive for *Haemoproteus* parasites survived less well over the next year. A possible explanation for this is that these infected individuals were suffering from a chronic infection, and thus already survived the acute and crisis stages of the infection where most mortality occurs^31^. This lack of an association between survival and infection status is consistent with the common pattern among studies on species suffering from infections with *Haemoproteus* blood parasites; correlative studies generally fail to detect any fitness consequences of infection, whereas medication experiments usually find an effect^19^. However, a recent study showed that while infections with *Plasmodium sp.* or *Haemoproteus sp.* experienced early in life may sometimes appear to have little or no immediate impact on fitness, these effects may only become apparent later in life, or as a cumulative effect over the lifespan^34^. In the great reed warbler (*Acrocephalus arundinaceus*), haemosporidian infections increased telomere shortening rate and were correlated with reduced lifespan^34^. Future studies may establish whether a similar mechanism occurs in the Seychelles warbler.

Apart from a cross-sectional study on long-lived mute swans (*Cygnus olor*), which also did not find an increase in the prevalence of *Haemoproteus* blood parasites in elderly individuals^15^, we are not aware of other studies in wild animals that tested for an increase in late-life haemosporidian parasite prevalence. We found no evidence that elderly individuals diagnosed with a *H. nucleocondensus* infection faced greater costs in terms of reduced survival to the next year than younger individuals. Although not entirely comparable (different type of parasite and host), this result contrasts with studies on *Plasmodium sp.* infections in elderly humans suggesting that immunosenescence contributes to higher intensity of *Plasmodium* infections and higher mortality of infected elderly patients compared with mid-aged patients^35^. Generally, studies that quantify the fitness costs of immunosenescence in the wild remain rare (but see ref. 11) and we encourage other studies to investigate late-life parasite prevalence and its fitness consequences in wild populations.

## Methods

### Ethics statement

All fieldwork was performed in accordance with local ethical regulations and agreements. The Seychelles Department of Environment and the Seychelles Bureau of Standards approved fieldwork and sampling. Nature Seychelles allowed us to work on Cousin Island Nature Reserve.

### Study species and data collection

The Cousin Island (29 ha; 4°20′ S, 55°40′ E) population of Seychelles warblers, comprising at any given time point *ca* 320 colour-banded adult individuals (of known age) in *ca* 115 territories, has been monitored since 1985 as part of a long-term study^17^,^36^. The Seychelles warbler’s life history is characterized by high annual adult survival (84%^37^) and low rates of reproduction (mostly single-egg clutches^25^). After hatching, nestlings remain in the nest for 18–20 days until they fledge and then receive parental care for up to three months^25^. The minimum age of first breeding is *ca* one year^25^. During the main breeding season in each year (June–September) from 1994–2015, the population was intensively surveyed for the presence of colour-banded individuals. As the annual resighting probability of adult individuals in this population is virtually one (0.98 ± 0.01 SE; 38), and because inter-island dispersal is rare^16^, we assumed that individuals that were not seen the next year were dead. For each individual that died before 2015, both the birth year and the death year were known.

Each year during the main breeding season, as many individual birds as possible were caught using mist nets and a blood sample (~25 μL) taken via brachial venipuncture. DNA was extracted using the methodology described by Richardson *et al*.^39^. Molecular sexing using a PCR-based method^40^ was used to confirm sex and to verify that extracted DNA from each blood sample was of suitable quality. All blood samples were screened for haemosporidian parasite infection (presence/absence) using a nested PCR technique following Hellgren *et al*.^26^. This method consists of an initial amplification of 20 cycles using the HaemNF1 and HaemNR3 primers, followed by a final amplification of 40 cycles using the HaemF and HaemR2 primers which bind to sites within the initial amplicon. This method amplifies a 479 bp section of the cytochrome *b* gene of *Haemoproteus* and *Plasmodium parasites.* PCR-based methods may sometimes fail to consistently amplify parasite DNA when it occurs at very low concentrations (within the host DNA extraction). Therefore, to reduce the possibility of false negatives, all samples were screened twice. Samples were recorded as being infected if they tested positive in either of the two independent PCRs (and no contamination was detected in each PCR using a ratio of 1 negative control for each 47 samples). Hutchings^23^ sequenced 40 positive samples collected over different years and found no other strains/types other than the GRW1 strain of *Haemoproteus nucleocondensus.*

Of the 1431 individuals that were screened for haemosporidian infections during the main breeding season (June–September), 852 individuals were screened in one year, 322 in two years, 144 in three years, 64 in four years, 32 in five years, 11 in six years, five individuals in seven years and one individual in nine years.

### Statistical analyses

#### Age-dependent Haemoproteus prevalence

To analyze age-dependent changes in *Haemoproteus nucleocondensus* prevalence (Y/N) we performed generalized additive mixed models (GAMMs) and generalized linear mixed models (GLMMs) with a binomial error structure and a logit link function. GAMMs^41^,^42^ are useful to investigate non-linear relationships, especially when the shape of the non-linear relationship is unknown. While in GLMMs a specific function needs to be specified to model a non-linear relationship between a dependent variable and a continuous predictor, GAMMs can account for non-linear relationships by including a non-parametric smoother. To control for non-independence of repeated observations of the same individual and differences in infection probability between years, individual identity and year were included as cross-classified random effects.

First, we determined and compared *Haemoproteus* prevalence in nestlings (<3 weeks old, *n *=* *269 samples), fledglings (3 weeks–3 months old, *n *=* *134), juveniles (3 months–1 year old, *n *=* *273) and adults (1–17 years old, *n *=* *1778).

Second, we investigated the overall age-dependent pattern of *Haemoproteus* prevalence in individuals of all ages (excluding nestlings and fledglings because infection may not yet be detectable in their blood), *n *=* *2051 samples from 1173 individuals) using a GAMM that included a non-parametric smoothing function for age, as well as the sex of the individual. Sex was included because a previous study on the Seychelles warbler found that males had higher *Haemoproteus* prevalence than females^14^. We then repeated this analysis using only individuals that had died before the end of the study (*n *=* *1512 samples from 891 individuals), and added age of death as a predictor to statistically control for potential selective disappearance of shorter-lived individuals with higher malaria prevalence^13^,^43^.

Third, we investigated linear within-individual changes in age-dependent malaria prevalence using within-subject centering^44^. Investigating such changes within individuals is important because cross-sectional patterns may differ markedly from within-individual patterns^13^,^44^. Because the GAMM analyses indicated a clear linear decline in *Haemoproteus* prevalence with age in individuals ≤4 years of age (see results), we first performed this within-subject centering analysis for all individuals aged 0–4 years. Then, in a separate analysis, we used this technique to investigate within-individual changes in individuals aged 7–17 years. This age range was chosen because 7 years marks the mean onset of senescence in this population^45^,^46^. In this way, we were able to explicitly study within-individual age-specific changes in *Haemoproteus* prevalence during early life and late life. For the within-subject centering we partitioned the age variable into a between-individual component – the mean age across all sampling events for each individual (hereafter: “mean age”) – and a within-individual component – the deviation in years from the individuals mean age for each sampling event (hereafter: “delta age”). In this analysis we excluded all individuals that were sampled only once. Mean age, delta age, and sex were included as predictors. A significant coefficient of delta age would indicate an age-specific change in *Haemoproteus* prevalence that occurs within individuals. We also tested the interaction between delta age and sex to test whether the within-individual change in age-dependent *Haemoproteus* prevalence differs between the sexes.

Finally, we performed an analysis on individuals that were screened for malaria infection in two consecutive years to estimate the age-dependent rate of loss and gain of *Haemoproteus* infections in infected and uninfected individuals, respectively^21^.

#### Haemoproteus infection and age-dependent survival

First, we performed GAMMs with binomial errors and a logit link function to investigate whether *Haemoproteus* infection predicted survival until one year later. We included infection status and sex, and a non-parametric smoothing term for age as predictors. As several individuals were screened in more than one year, individual identity was included as a random effect following e.g. refs 47,48. In addition, year was included as a random effect. We then repeated this analysis for younger individuals (≤4 years old) and older individuals (≥7 years old) separately using GLMMs. In addition to infections status, sex, and age, we also included the interaction between infection status and age in these analyses to investigate whether the impact of *Haemoproteus* infections on survival changed with age.

#### Model selection

We performed all analyses using R version 3.2.3 (R Development Core Team 2015). The GLMMs were performed using the package lme4 version 1.1.10 and GAMMs using the package gamm4 version 0.2–3. Final models included all main effects (irrespective of their significance) and any significant (*P* < 0.05) two-way interactions (see ref. 49). Non-significant two-way interaction terms (*P* > 0.05) were removed from the models. Results from models containing only significant predictors were identical.

## Additional Information

**How to cite this article**: Hammers, M. *et al*. Age-specific haemosporidian infection dynamics and survival in Seychelles warblers. *Sci. Rep.* **6**, 29720; doi: 10.1038/srep29720 (2016).

## Figures and Tables

**Table 1:** Prevalence of infections with the GRW1 strain of *Haemoproteus nucleocondensus* in relation to age in Seychelles warblers that were (a) 0–4 years of age and (b) 7–17 years of age.

*Haemoproteus* prevalence		β	SE	*z*	*P*
(*a*) *Individuals* ≤*4 years*	Intercept	1.23	0.40	3.10	<0.01
	Mean age	−0.85	0.15	−5.68	<0.001
	Delta age	−1.01	0.12	−8.68	<0.001
	Sex	0.33	0.20	1.65	0.10
	Sex : delta age	−0.13	0.18	−0.75	0.46
	Random	Variance			
	Bird identity	0.79			
	Year	1.21			
(*b*) *Individuals ≥7 years*	Intercept	−3.07	1.56	−1.97	0.05
	Mean age	0.12	0.15	0.79	0.43
	Delta age	−0.07	0.12	−0.56	0.57
	Sex	0.65	0.47	1.37	0.17
	Sex : delta age	−0.07	0.26	−0.28	0.78
	Random	Variance			
	Bird identity	0.56			
	Year	<0.01			

Mean age is the change in infection prevalence with age occurring at the population level and delta age is the within-individual age-dependent change in prevalence.
